# Supplementary material for: Exploring the Role of Communication Asset Mapping (CAM) as a Strategy to Promote Hereditary Cancer Risk Assessment Information Within African American Communities
Source: Int J Environ Res Public Health. 2025 Jan 8;22(1):75. doi: 10.3390/ijerph22010075 (PMC11771212; doi:10.3390/ijerph22010075)
Supplement: Supplementary file 1 [file ijerph-22-00075-s001.zip › ijerph-3218423-supplementary.pdf]

Communication Asset Mapping Project

Workshop Facilitator Guide

Prior to the start of the workshop, all participants will be given information about the study and asked to sign informed consent forms. They will be provided with answers to any questions they may have and all aspects of participation and informed consent will be explained. Anyone who does not wish to participate or will not sign a consent form will not be permitted to participate. All workshops will be led by a community facilitator, assisted by an assistant facilitator (Community Advisory Board member). The assistant facilitator or designated note taker will take notes while the facilitator leads the group.

This is intended as a guide for the workshop facilitator or assistant facilitator. As each workshop group progresses, follow-up questions may be asked on any of the topics discussed. Questions will flow from one to the next guided by the group. Participants will be informed that they may choose not to answer any questions that they are uncomfortable answering prior to the start of the Workshop session. Topics not included in this guide will not be brought up by the workshop group facilitators. This guide is intended for use by the facilitators and will focus on understanding identifying community resources to develop a Communication Asset Map for African American faith populations as it relates to Cancer-related Genetic Counseling and Testing health promotion. Workshop group participants will include self-identified African Americans in faith populations who are age 18 and older. Workshop group participants will each receive a $50 gift card.

****

Thanks again for agreeing to participate in this workshop. We're audio-taping this workshop and it will be transcribed. We will not put your name on the transcript and will not use your name in conjunction with any quotes we may use. We are very interested in your thoughts about community locations that African Americans in faith communities would receive cancer prevention information (for example community clinics, pantries, grocery stores, salons, banks etc.). Our ultimate goal is to develop a Communication Asset Map focused on cancer-related counseling and testing information for Africans Americans who are a part of or affiliated with faith-based organizations. This Communication Asset Map will detail locations that are areas that African Americans within faith communities frequent and are places that they could retrieve or receive trusted information about cancer prevention. This Communication Asset Map will include assets that are near faith-based organizations within the Faith Works Connecting for a Healthy Community network and bridge these populations to Cancer-related genetic counseling and testing health information. Ultimately, the information would be used by lay health educators within faith communities and could be beneficial for those living in these communities and those who are affiliated with these communities.

Before we start the workshop, we want to confirm that each of you has consented to participate in this study and have filled out a demographic questionnaire that was emailed to you via our RedCap secure link. The survey includes questions about basic demographic information, such

as your age, education, and church affiliation and your cancer history. If you have not consented into the study, we will have you do that now and complete the survey.

Check in for Survey Completion -

****
I'd like for all of us to now talk as a group. This workshop which is primarily a participatory discussion about building a Communication Asset Map for faith communities, should last approximately one hour. First, we'll talk about the format; second, we will identify assets based on your experiences within your faith community. Last, we will then talk about the accompanying communication map. Does anyone have any questions before we get started?
****

Prior to start: Technology support will pull up the asset map virtually for all of the participants to see. *Each of the participants also will have received a copy of the map at least 2 days prior.* Allow for each of the participants to orient themselves with the map again prior to the start of the community workshop. It will be the responsibility of the notetaker to take notes on the entire process.

1. **Format (Est. 40-45 minutes)**
   - **Overview**
     - We're going to: Define assets
     - Review some of the places in the community that our Community Advisory Board (CAB) has identified that are places that African American faith populations go the most; from this list, we will then rank them (For this workshop, we are focusing on two of the Faith Works affiliate churches – one is in Kansas and the other in Missouri)
     - We will then discuss whether you agree that these are the community assets
     - What's in the community? (assets and non-assets)
     - What's missing?
2. **Mapping Introduction (5 Minutes)**
   a. "Can anyone tell me what an asset is?" (Technology/Notetaker individual records the responses to this question on the electronic white board) (assets - a useful or valuable thing, person, or quality)
      i. After recording several responses, summarize them, and, if not already stated, ask participants if you can all agree that an asset is "a positive place or program that makes the community a safe, healthy, and good place to live."
      ii. Notetaker/Technology person will add the definition to the electronic white board or chat box

iii. Facilitator: "When we talk about 'assets' during this exercise, think about these definitions that we've discussed here." (other Faith-based organizations in the area)

3. **<u>Review Asset Mapping Process and Review Directions/Example (5 Minutes)</u>**

   a. To get a sense of the assets that are in the community that we designated in Missouri and Kansas and that are assets to the churches (Palestine Missionary Baptist Church and Bethel Baptist Church) in our designated areas in the context of cancer-related genetic counseling and testing, we want to know about the places that ***you*** think are assets that are near and around this area, and whether or not you think they're assets. ***We have already identified some with our Community Advisory Board but we also welcome additional assets that you can think of.***"

      i. Each participant will <u>vote</u> **<u>via Zoom polling at the end or when we all reconvene in the large Zoom room (we will email all participants the polling sheet/form)</u>**

   b. "I will now show you a document that will detail the places that our Community Advisory Board has identified as places that are communication assets and therefore places that would be good to have information about Cancer-Related Genetic Counseling and Testing communication within the community. There are multiple places with questions." **(They should have received a copy).**

   c. (Direction for Facilitator – not read aloud to the participant) **Look at the example on your poll. Have someone read the question: Where do you go for preventive health (or where would you think you can get information that you can trust or trusted information)?**

      i. **NOTE: Make sure that you remind the participants that the communication resources should also have good assets or people within the location (for example Panera Bread – it is a place to get health food but it may be too much or perceived to be expensive)**

      ii. **There may be other places that they would recommend that have not been identified (for example Popeye's chicken, etc.)**

   d. "For each community resource/asset listed on this document, think about how familiar you are with this area and where people that are affiliated with these faith-based organizations go. **Please keep in mind that we will ask you to rate these in the larger group after we leave this break-out session**. Some of those questions are the following:"

      i. "Where does a person in this area go for other health and wellness activities and where is it located?" (**Pull up the identified assets and map**)

      ii. "When you get to the larger group, you will decide on the assets that you think would be best and you will also respond to *why* you selected it. Here in the break-out group, I would encourage you to think about how you would also rank these based on a 5-point scale rating point system (for

example, how you would rate a restaurant or a hotel if it is a 3-star, 4 star etc.) 5 means “I think it’s a very good asset and it’s the best; 3 means average and that “I go there but I don’t think it’s a strong asset.” 1 is very poor and I would not consider it as an asset, and I wouldn’t recommend it at all. Remember the discussion we had on what an asset is in terms of the faith community (designated church).”

iii. “While you will not be able to rank this now but in the larger group, this will be a way to think about how you select the assets when you respond to the poll in the larger group. Does anyone have any questions?”

e. “Once we have gone through all of the questions, we’ll go back and look at all of the points on the map also.” Let’s go through them.” **(Make sure to have each person participate.)**

4. **<u>Discuss Existing Assets (on the Map identified by CAB) and Missing Assets? (10-15 minutes) (We Will Go Over This Again In The Larger Group)</u>**

a. **Facilitators will lead the conversation here, but it can be conversational – we will revisit this in the larger group**

b. Questions to help facilitate the dialogue:

i. What do you think about the communication assets identified in the designated areas near (Palestine Missionary Baptist Church/Bethel Church)?

ii. **Participants will review the map and the identified areas**: Are there particular clusters of the places that they go to? Clusters of specific types of places? Clusters of grades (clusters of 5’s means clusters of assets, clusters of 1’s means clusters of non-assets)? If so, why do they think these clusters exist? Are there specific intersections and locations where their places/assets are located? Write down any specific geographic locations to the questions below.

iii. Notetaker/Scribe will record participants’ response to the questions below on the white board, record location as well when possible.

iv. **Assets in the Community (5 star, 4 star to 3 star – look at these)**

1. **What:** Of the places on the map, which places are the best assets? Any missing?
2. **WHY:** Why do you consider these places assets?
3. **What about Location:** Is there something about where they’re located that makes them an asset?
4. **Non-Assets – in the community**
5. **What:** Of the places on the map, which places ARE NOT assets to you that have been identified?
6. **Why:** Why don’t you consider these places assets?

7. **What about location:** Is there something about where they're located that would make you say they're not an asset?
8. **What can be improved?**
    a. Of all the places on the map, are there places that can be improved to become assets or become better assets? What kind of improvements?
9. **WHAT'S MISSING? (SEE Questions Below to expand on your question)**

c. **Facilitator: Notice the places that people go outside of the community to access.**
1. **ASK:** Looking at the places that people from (Palestine or Bethel) or affiliated with these Faith-Based Organizations go to outside the community/area, would you say that these places are assets missing from the community? What types of these assets are missing?
2. **WHAT ELSE?** Are there even other types of programs or places that are missing from the community?

ii. "How did you grade the locations that you deemed as the best assets (location?).

iii. **Facilitator (if time) can ask for participants to discuss their selections or any additional assets that were not brought up**

iv. **REMINDER for Facilitator to leave the room around 7 p.m.**

5. **<u>LARGE ROOM DISCUSSION (20-25 Minutes) – (Transition) 6:55-7 p.m.</u>**
    a. **Brief summary from all break out groups**
    b. **Primary Facilitators (Crystal, Garry and Howard) will then go through each question with ALL participants. For each question:**
        i. **Facilitator reads the question aloud**
        ii. **Participants will respond to polls through zoom (Garry or Howard to manage).**
        iii. **Technology will Project the completed Zoom poll results.**
6. **<u>Discuss Existing Assets (on the Map identified by CAB) and Missing Assets? (10-15 minutes)/Polling</u>**
    a. Crystal and Facilitators will lead discussion here
    b. Questions to help facilitate the dialogue:
        i. What do you think about the communication assets identified in the designated areas near (Palestine Missionary Baptist Church/Bethel Church)?

ii. **Participants will review the map and the identified areas**: Are there particular clusters of the places that they go to? Clusters of specific types of places? Clusters of grades (clusters of 5's means clusters of assets, clusters of 1's means clusters of non-assets)? If so, why do they think these clusters exist? Are there specific intersections and locations where their places/assets are located? Write down any specific geographic locations to the questions below.

iii. Notetaker/Scribe will record participants' response to the questions below on the white board, record location as well when possible.

iv. Assets in the Community (5 star, 4 star to 3 star – look at these)

1. **What:** Of the places on the map, which places are the best assets? Any missing?
2. **WHY:** Why do you consider these places assets?
3. **What about Location:** Is there something about where they're located that makes them an asset?
4. **Non-Assets – in the community**
5. **What:** Of the places you put on the map, which places ARE NOT assets to you that have been identified?
6. **Why:** Why don't you consider these places assets?
7. **What about location:** Is there something about where they're located that would make you say they're not an asset?
8. **What can be improved?**
   a. Of all the places on the map, are there places that can be improved to become assets or become better assets? What kind of improvements?
9. **WHAT'S MISSING? (SEE Questions Below to expand on your question)**

7. <u>Wrap-Up Discussion (Summarize Process) (5-10 Minutes)</u>

a. Briefly summarize their feedback. Particularly, review maps with the group to identify:

i. Any particular clusters of assets

ii. Any gaps/areas without any assets

b. Polls tabulated and notetaker notes collected to store electronically (submit to designated KUMC staff person who will place on the P Drive)

c. Look at the map of the area (will be emailed to individuals and will be shown virtually). The map includes the areas that the churches are located (Palestine Missionary Baptist Church and Bethel). We will ask you about different aspects of the map and what you think about the identified areas. What did you think about this process?

8. Take a look at the areas on the map. What do you think of them? (End Here)

Once again, thanks so much for your time today. Through this community workshop, we hope to learn communication and educational tools that will help increase awareness about cancer-related genetic counseling and testing among African Americans. Does anyone have anything they want to ask us? Thanks again! Please also be sure that we have your attendance to email or mail you your $50 gift card.
